# Effects of Posture and Stimulus Spectral Composition on Peripheral Physiological Responses to Loud Sounds

**DOI:** 10.1371/journal.pone.0161237

**Published:** 2016-09-01

**Authors:** Jennifer Koch, Jan Flemming, Thomas Zeffiro, Michael Rufer, Scott P. Orr, Christoph Mueller-Pfeiffer

**Affiliations:** 1 Department of Psychiatry and Psychotherapy, University Hospital Zurich, Zurich, Switzerland; 2 Neural Systems Group, Massachusetts General Hospital, Boston, MA, United States of America; 3 Department of Psychiatry, Massachusetts General Hospital and Harvard Medical School, Boston, MA, United States of America; Universidad de Salamanca, SPAIN

## Abstract

In the “loud-tone” procedure, a series of brief, loud, pure-tone stimuli are presented in a task-free situation. It is an established paradigm for measuring autonomic sensitization in posttraumatic stress disorder (PTSD). Successful use of this procedure during fMRI requires elicitation of brain responses that have sufficient signal-noise ratios when recorded in a supine, rather than sitting, position. We investigated the modulating effects of posture and stimulus spectral composition on peripheral psychophysiological responses to loud sounds. Healthy subjects (N = 24) weekly engaged in a loud-tone-like procedure that presented 500 msec, 95 dB sound pressure level, pure-tone or white-noise stimuli, either while sitting or supine and while peripheral physiological responses were recorded. Heart rate, skin conductance, and eye blink electromyographic responses were larger to white-noise than pure-tone stimuli (p’s < 0.001, generalized eta squared 0.073–0.076). Psychophysiological responses to the stimuli were similar in the sitting and supine position (p’*s* ≥ 0.082). Presenting white noise, rather than pure-tone, stimuli may improve the detection sensitivity of the neural concomitants of heightened autonomic responses by generating larger responses. Recording in the supine position appears to have little or no impact on psychophysiological response magnitudes to the auditory stimuli.

## Introduction

Stress sensitization of the nervous system is believed to play an important role in the development of posttraumatic stress disorder (PTSD) after a traumatic event and may underlie symptoms such as hypervigilance and exaggerated startle [1]. Sensitization of the autonomic nervous system in PTSD is evidenced by heightened cardiovascular and, less consistently, electrodermal and electromyographic responses to startling sounds in trauma survivors who develop PTSD [2–5]. Prospective and twin studies have indicated that increased autonomic reactivity to startling sounds is an acquired PTSD sign rather than a pre-existing risk factor, and may thereby serve as a measure of stress sensitization [4,6].

The neural basis of autonomic stress sensitization is a largely neglected area in PTSD research. The loud-tone procedure [7] involves presenting a series of 15, long-duration (500 msec), 1000 Hz, 95 dB sound pressure level (SPL), sudden onset pure tones in a task-free situation. It has become an established paradigm in PTSD research for investigating autonomic stress sensitization. Successful use of this procedure during functional magnetic resonance imaging (fMRI) requires the elicitation of brain responses that are of sufficiently large amplitude to be detected against a background of physiological noise. Moreover, differences between the psychophysiological laboratory and neuroimaging environments, such as background noise and the subject’s postural position, require consideration.

In a previous study employing the loud-tone procedure during fMRI in healthy subjects, we found a significant association between neural activity and skin conductance (SC), but not heart rate (HR), responses to the pure tones [8]. However, our failure to detect neural concomitants of HR responses may have reflected Type II error. The use of stimuli that are capable of generating larger neural and psychophysiological responses may help to optimize fMRI detection sensitivity and thereby reveal underlying neural mechanisms. Previous research has shown larger HR [9], SC [10] and EMG [11] responses to white noise compared to pure tones, and a positive association between the magnitude of these responses and stimulus intensity [11–16].

Although the background noise of the scanner can be partially reduced by the use of active sound-cancellation systems, there is a potential effect of supine versus sitting position on peripheral autonomic responses. Previous research has suggested that resting HR level is higher in the sitting than supine position [17–19]. However, it is not clear whether HR responses to stimuli such as loud sounds are influenced by posture. Moreover, we are not aware of research that has examined the effect of posture on SC and eye-blink EMG responses.

In this study, we investigated modifications to the loud-tone procedure for optimizing the procedure for use during fMRI. Specifically, we investigated how HR, SC and eye-blink (left orbicularis oculi) EMG responses were influenced by stimulus spectral bandwidth (pure tone vs. white noise) and subject’s posture (sitting vs. supine). We hypothesized that white noise stimuli would induce larger autonomic and eye blink responses than pure tones [11]. Given previous findings of higher resting HR level in the sitting position [17–19], we expected to observe larger HR responses to the auditory stimuli in the sitting, compared to supine, position.

## Methods

### Subjects

Subjects were 24 medication-free individuals, recruited by advertisement. Demographic information is presented in Table 1. All participants were free of a psychiatric, major medical or neurological illness, including current or past traumatic brain injury and substance dependence according to self-reports.

**Table 1:** Demographic and Psychometric Sample Characteristics (*N* = 24).

	*Mean*	*SD*
Age (Years)	24.12	4.66
Education (Years)	15.38	2.90
BDI	6.04	2.88
BSI: Global Severity Index	0.30	0.20
STAI: Trait Anxiety	33.25	5.12
	***N***	***%***
Sex: Female	17	70.8
Marital Status: Single	24	100

SD: Standard deviation; BDI: Beck Depression Inventory; BSI: Brief Symptom Inventory; STAI: State-Trait Anxiety Inventory.

Subjects completed the Beck Depression Inventory (BDI) [20,21], Brief Symptom Inventory (BSI) [22,23], and the Trait portion of the State-Trait Anxiety Inventory (STAI) [24,25]. All measures had been previously translated into German and validated. Psychometric information is presented in Table 1. The study protocol was approved by the Institutional Review Board of the Canton of Zurich, Switzerland. All participants provided written informed consent according to the Helsinki Declaration.

### Task Procedures

Experimental procedures were similar to those used in previous studies of PTSD [2–4,6,26–31]. An audiometric test was performed monaurally using a Midimate 602 Diagnostic audiometer (Madsen Electronics, GN Otometrics A/S, Denmark) and included 125, 250, 500, 1000, 2000, 4000, and 8000 Hz tones. In order to be enrolled in the study, subjects were required to detect 25 dB hearing level tones in both ears and to show an observable change in SC level to a startling sound or psychological stressor (calculation task). Stimuli were created using Audacity software (http://audacity.sourceforge.net) and consisted of 500 msec, 95 dB SPL, pure tones (1000 Hz) or white noise (5 – 22K Hz). The rise and fall times of stimulus onset and offset were set to 0 msec according to the selected software setting. Actual rise and fall times as measured from the amplifier were 1 msec, and less than 1 msec, respectively. The inter-trial interval varied between 27 and 52 sec. Stimuli were presented binaurally through circumaural earphones (PD-81, Novitronic Inc., Zurich, Switzerland).

Subjects underwent four experimental conditions that varied with respect to stimulus type (pure tone or white noise) and posture (sitting or supine). The four conditions were pseudo-randomized and counter-balanced across subjects. Each experimental condition was separated by 4–16 days (median inter-session interval = 7 days, interquartile range = 1.00). During the laboratory assessment, subjects were instructed to keep their eyes open and not to move while listening to the sounds. The auditory stimuli were presented following a 5 min resting baseline period.

In a separate task, the perceived loudness and valence of the sounds were investigated using magnitude estimation, which is an established procedure to measure perceived loudness of sounds [32]. Subjects were asked to assign an individual number to a 65 dB SPL pure tone presented as reference. Then, 35, 50, 65, 80, or 95 dB SPL pure tones or white noise sounds were presented in randomized order and subjects were asked to assign a rating for each sound relative to the value of the initial reference sound reflecting perceived loudness (higher = louder) and valence (higher = more aversive). Each combination of sound type and sound level was presented three times. Sound loudness and valence were estimated in separate runs. Sound pressure levels were determined using a Voltcraft SL-100 sound level meter (Conrad Electronic AG, Wollerau, Switzerland) and measured at the headphone’s output. The procedure was implemented using E-Prime Professional 2.0 (Psychology Software Tools, Inc., Sharpsburg, PA).

### Data Acquisition

Psychophysiological data were obtained with a Biopac modular instrument system (Biopac Systems, Inc., Goleta, CA). Cardiac interbeat intervals were recorded with a biopotential amplifier (EOG100C) using pre-gelled Biopac electrodes (EL503). The tachometer output was updated with each successive heartbeat and provided a continuous measure of HR (in beats per minute, BPM) that reflected the immediately preceding interbeat interva and. SC was recorded with an MRI-compatible electrodermal activity amplifier (EDA100C-MRI) using disposable radiotranslucent Biopac electrodes (EL509) filled with isotonic gel placed on the hypothenar surface of the participant's nondominant hand. Eye-blink EMG was recorded by an electromyogram amplifier (EMG100C) using silver-silver chloride electrodes (EL254). For EMG, the skin was swabbed with an alcohol pad, and electrodes were filled with electrolytic gel and placed over the right orbicularis oculi. Psychophysiological analog signals were digitized at 1000 Hz by an analog-to-digital converter (MP150). Analog amplifier cut-off frequencies were 1 Hz for the EDA100C-MRI, 0.05Hz high-pass and 35 Hz low-pass for EOG100C, and 100 Hz high-pass and 500 Hz low-pass for the EMG100C. Gain was 10uOhm/V for EDA100C-MRI and 2000 for EOG100C and EMG100C.

### Scoring of psychophysiological data

Psychophysiological data processing followed previously published methods [4]. Although alternative methods may be reasonable, there is a strong precedent given the extensive literature regarding measuring autonomic sensitization in PTSD using these parameters [2–4,6,26–31].

HR and SC response scores were calculated for each stimulus presentation by subtracting the mean level during the 1 sec interval preceding stimulus onset from the highest level within the 1–4 sec interval following stimulus onset. EMG response scores were calculated by subtracting the average EMG level for the 1-sec interval preceding stimulus onset from the maximum level within 40 to 200 msec of stimulus onset. The raw EMG signal was rectified and integrated using a 10-msec time constant. Artifactual HR and SC responses were excluded by visual inspection of each subject’s data. Square root transformations were performed for all responses to reduce heteroscedasticity.

For HR, SC and EMG, an averaged response score was calculated for each subject for the transformed responses to the 15 stimulus presentations within each condition. A measure of relative habituation for HR, SC and EMG responses was calculated from the slope of the regression equation *Y = a + bX* for trials 2–15, where *Y* represented the square root of the response score and *X* represented the log trial number. Locally weighted scatter plot smoothing (LOESS) was used for fitting nonlinear, piecewise polynomial curves for SC and HR responses, as we have previously done [8].

### Statistical analysis

The influence of bandwidth and subject's posture on averaged psychophysiological response scores and slope scores (the measure of relative habituation) were analyzed with a series of repeated measures analyses of variance (ANOVAs) with bandwidth (pure tone, white noise) and subject’s posture (sitting, supine) as within-subject factors. An average magnitude estimation score was calculated for each sound type and sound pressure level across the three presentations. We used a linear mixed-effects model design to analyze the effect of sound pressure level (dB) and sound spectral bandwidth (pure tone, white noise) on log-transformed sound loudness and valence estimation scores. Sound pressure level and sound bandwidth were treated as fixed effects and subject as random effect.

The critical threshold was p *<* 0.05. We used R V.2.14.1 for statistical analyses [33].

## Results

**Fig 1:**
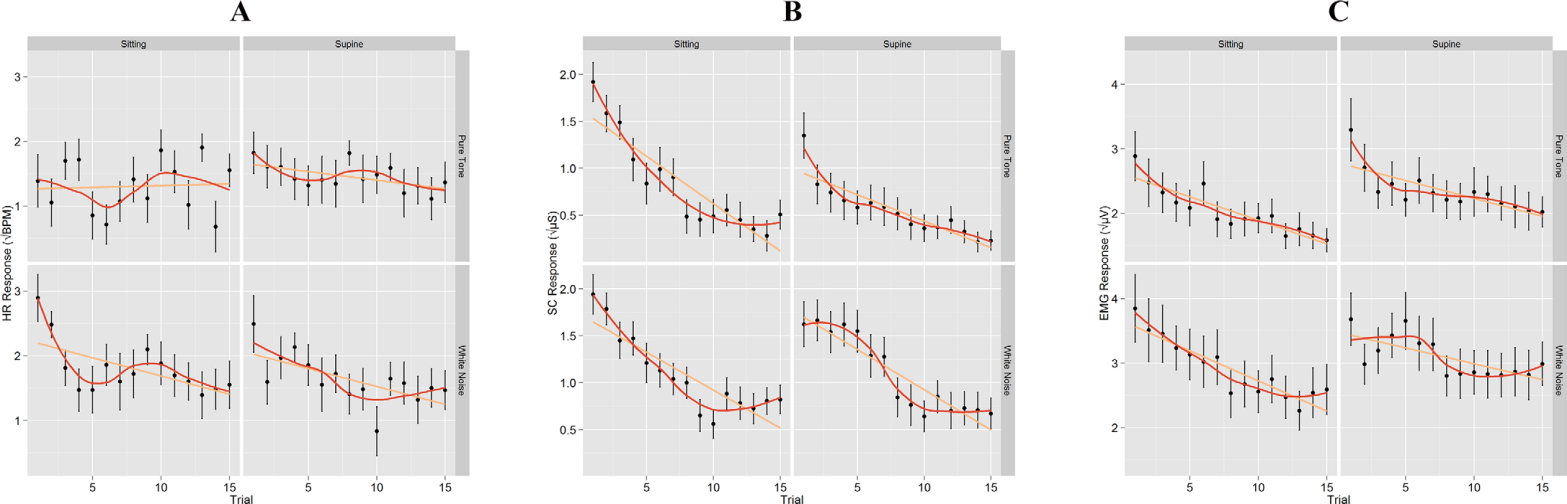
Group means for time course of HR (A), SC (B), and eye blink EMG (C) responses to sound presentations by stimulus bandwidth and subject’s posture.

HR, SC, and EMG responses to 95 dB SPL white noise stimuli were larger than to 95 dB SPL pure-tone stimuli as evidenced by significant main effects for bandwidth (p’*s* < 0.001, η2G’*s* ≥ 0.073; Fig 1, Table 2). Posture did not have a significant effect on psychophysiological responses (all p’*s* ≥ 0.082; Table 2). As can be seen in Table 2, HR and SC resting levels were higher when subjects were in sitting, compared to supine as evidenced by significant main effects of posture for pre-tone HR and SC levels (*p*’s ≤ 0.014, all η2G’*s* ≥ 0.035). Posture had no effect on EMG pre-tone level (*p* = .174).

**Table 2:** Peripheral Physiological Responses to Sound Presentations by Stimulus Bandwidth and Subject’s Posture (N = 24).

	95 dB SPL Pure Tone				95 dB SPL White Noise				ANOVAa								
	Sitting		Supine		Sitting		Supine		Bandwidth			Posture			Bandwidth x Posture		
HR	*Mean*	*SD*	*Mean*	*SD*	*Mean*	*SD*	*Mean*	*SD*	*F*	*p*	*η2G*	*F*	*p*	*η2G*	*F*	*p*	*η2G*
Pre-Tone Level (BPM)	73.73	12.19	68.08	9.59	70.95	11.19	65.41	9.58	6.64	.017	0.017	17.37	.000	0.067	0.004	.949	0.000
Mean Response (√ BPM)	1.31	0.59	1.45	0.56	1.79	0.62	1.63	0.64	16.58	.000	0.074	0.002	.969	0.000	2.614	.120	0.016
Response Slope	0.01	0.10	-0.03	0.08	-0.05	0.12	-0.06	0.09	7.89	.010	0.044	0.964	.336	0.009	0.439	.514	0.004
SC																	
Pre-Tone Level (μS)	14.19	7.92	10.20	9.27	14.96	8.99	12.69	7.31	1.61	.217	0.010	7.129	.014	0.035	0.376	.546	0.003
Mean Response (√ μS)	0.84	0.67	0.55	0.71	1.08	0.59	1.10	0.83	14.92	.000	0.076	1.741	.200	0.010	2.264	.146	0.012
Response Slope	-0.10	0.07	-0.06	0.06	-0.08	0.05	-0.09	0.07	0.358	.556	0.002	2.003	.170	0.025	5.904	.023	0.038
Eye Blink EMG																	
Pre-Tone Level (μV)	0.67	0.05	0.77	0.36	0.70	0.09	0.68	0.07	0.937	.343	0.010	1.97	.174	0.024	6.275	.020	0.048
Mean Response (√ μV)	2.04	1.14	2.34	1.35	2.91	1.72	3.09	1.63	25.64	.000	0.073	3.297	.082	0.007	0.173	.682	0.000
Response Slope	-0.07	0.09	-0.06	0.06	-0.09	0.10	-0.05	0.05	0.254	.619	0.002	4.79	.039	0.039	1.865	.185	0.008

df’s = 1, 23. All mean values are significantly different from zero (p < .005), except for HR response slopes for pure tones in sitting and supine position (p’s ≥ .144) and slope of HR responses to white noise in sitting position (p = .058). SD: Standard deviation; HR: Heart rate; SC: Skin conductance; EMG: Electromyogram; BPM: Beats per minute; μS: Micro Siemens; μV: Micro Volt.

Relative habituation of HR responses to the white noise stimuli was observed for the supine position (*t*(23) = -3.21, *p* = .004; Fig 1A) and for SC and EMG responses for both bandwidths and postures (*p*’s ≤ .001; Fig 1B and 1C).

**Fig 2:**
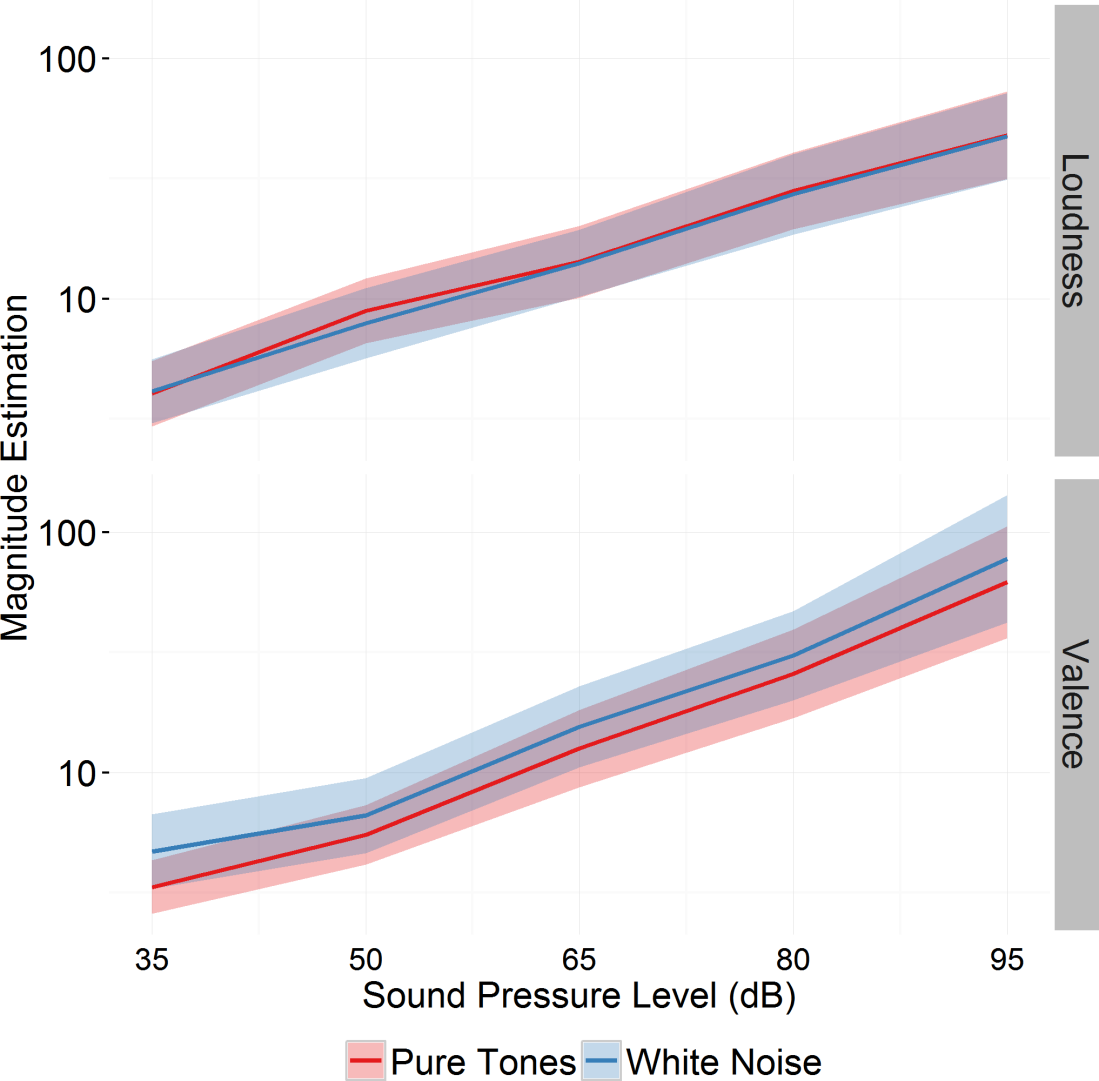
Magnitude estimation of loudness and valence of pure tones and white noise relative to a reference stimulus (65 dB SPL pure tone). Higher scores represent louder and more aversive perception. Relative loudness of sounds (as represented by the slope of the curves) did not differ between stimulus types. The ribbon represents standard errors. Data were available from 15 subjects.

Subjects experienced sounds of higher sound pressure levels as louder (*t*(135) = 14.66, *p* < .001) and more aversive (*t*(150) = 9.27, *p* < .001; Fig 1A). There was no significant main effect of sound type or a sound type by sound pressure level interaction effect for perceived loudness (*t*(135) = -0.14, *p* = .888 and *t*(135) = 0.04, *p* = .970) and aversiveness (*t*(150) = 0.59, *p* = .555 and *t*(150) = -0.23, *p* = .821; Fig 2).

## Discussion

In this study, we investigated the effect of two important modifications to the loud-tone procedure, for their potential impact on recording during fMRI. In line with our predictions, we observed larger HR, SC and eye blink EMG responses to the white noise, compared to pure tone, stimuli. Physiological responses to the sounds were found to be comparable in the sitting and supine position, contrary to our expectations. The finding of larger responses to the white noise, compared to pure tone, stimuli is in agreement with previous studies that found larger HR [9], SC [10], and EMG [11] responses to white noise stimuli. These findings confirm the proposition that the magnitude of autonomic and startle responses are influenced by stimulus spectral bandwidth [34]. Using white noise, rather than pure tone, stimuli in the “loud-tone” procedure during fMRI may enhance the detection sensitivity for future investigations of neural correlates of autonomic sensitization in PTSD.

There is ample evidence for higher resting HR in the sitting, compared to supine, position [17–19], which is in line with our results. However, in contrast to the different HR levels, we observed comparable HR response magnitudes to the stimuli when subjects were tested in the sitting and supine positions. This suggests that HR responses associated with the immediate reaction toward a sudden environmental change [34,35], as assessed and scored in this study, are not substantially influenced by hydrostatic pressure, sympathetic tone changes or other factors that change with body posture.

Participants in this study had higher resting SC levels while sitting, compared to supine. This finding is consistent with characteristics of palmar activity of sweat glands, i.e., greater spontaneous sweat release in sitting versus supine position [36]. Sweat secretion is correlated with electrodermal activity [37].

Our study has several limitations. First, the results are based only on healthy subjects; consequently, we do not know whether the suggested modifications of the “loud-tone” procedure would reliably discriminate individuals with PTSD from trauma-exposed healthy controls, because the white-noise stimulus may be sufficiently potent to obscure reactivity differences because of ceiling effects. Alternatively, the more provocative white-noise stimulus may serve to enhance previously observed group differences. Second, other characteristics of the fMRI environment such background noise and confinement stress were not investigated in this study, which was not conducted in a scanner. Third, although active noise-cancellation systems can reduce scanner noise by approximately 30 dB SPL, autonomic responses to sound stimuli might still be confounded by background noise. Finally, we did not control internal factors such as body temperature and climatic conditions (e.g, ambient air temperature, humidity and pressure), which have been shown to influence SC [34].

In conclusion, our results indicate that supine positioning, as currently necessary for MRI, does not substantively change the characteristics of autonomic responses during the loud-tone procedure. Using broadband sounds, such as white noise instead of pure tones, will serve to increase autonomic response magnitudes and thereby could enhance our ability to detect significant associations with neural responses. A modified version of the loud-tone procedure might be suitable for future fMRI studies investigating autonomic sensitization in PTSD, and possibly other disorders.

## Supporting Information

S1 Dataset The psychometric data. (SAV)

S2 Dataset The psychophysiological data. (CSV)

S3 Dataset The magnitude estimation data. (CSV)
